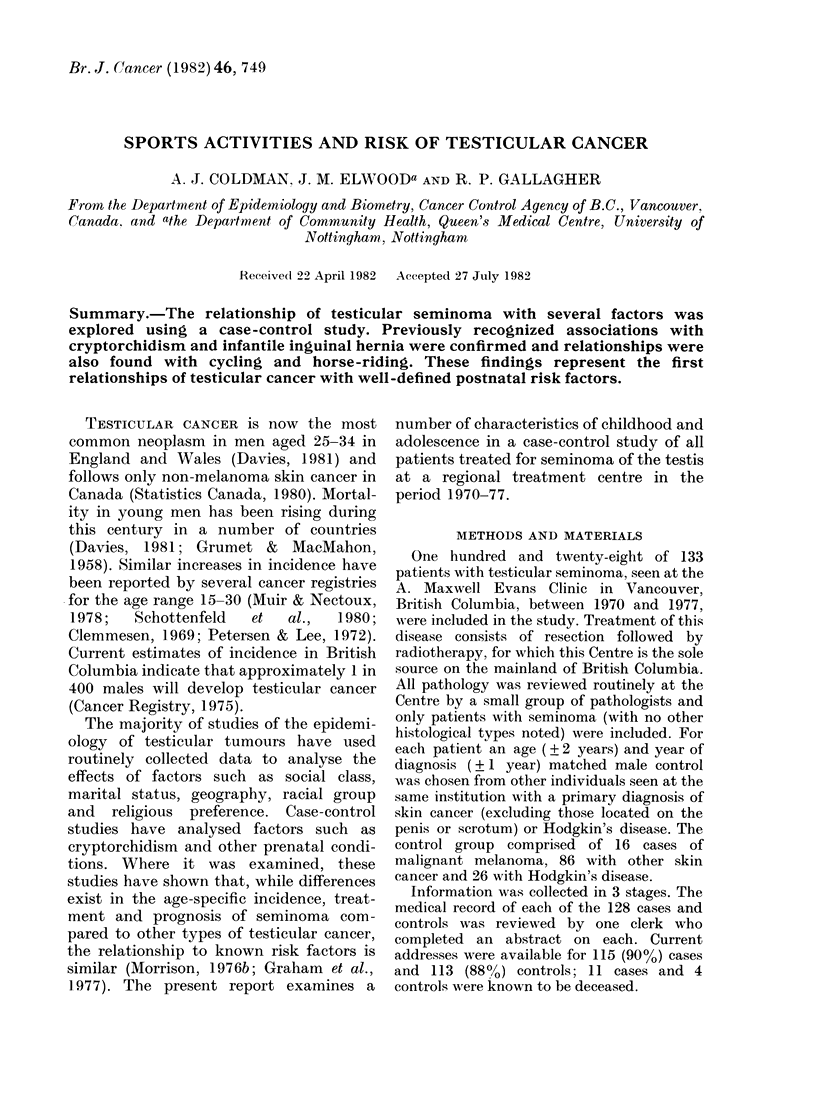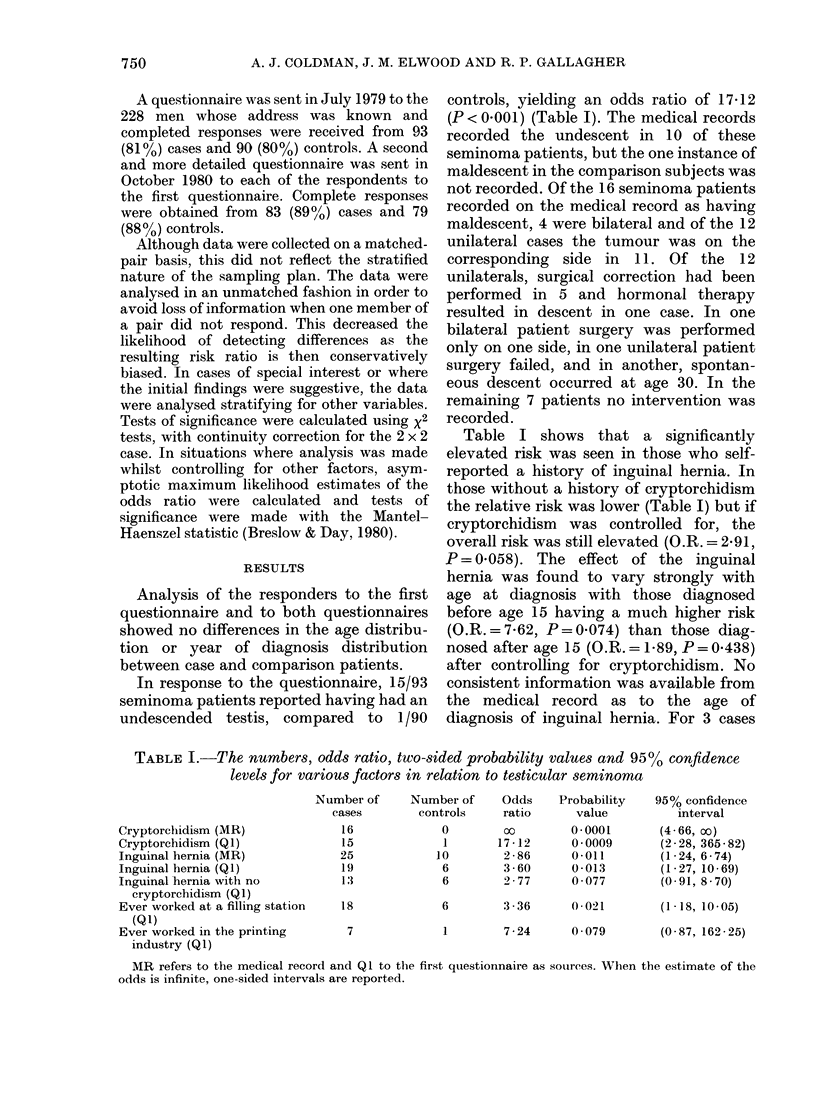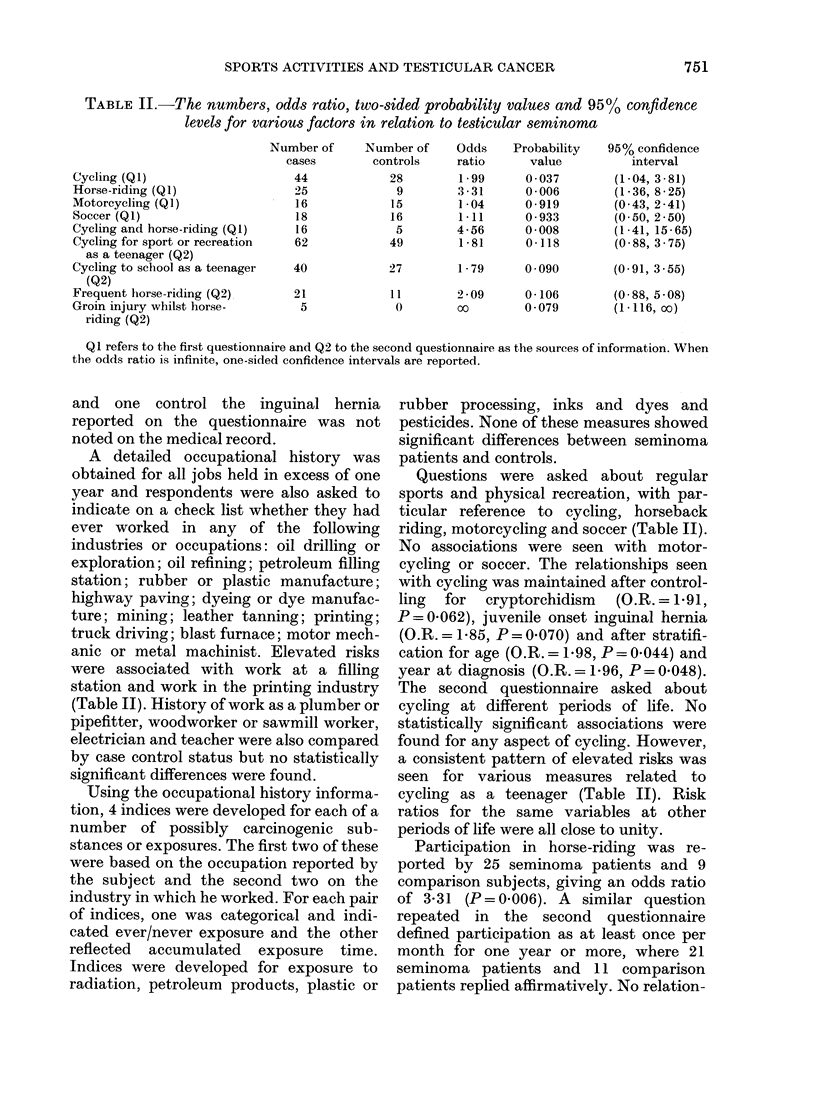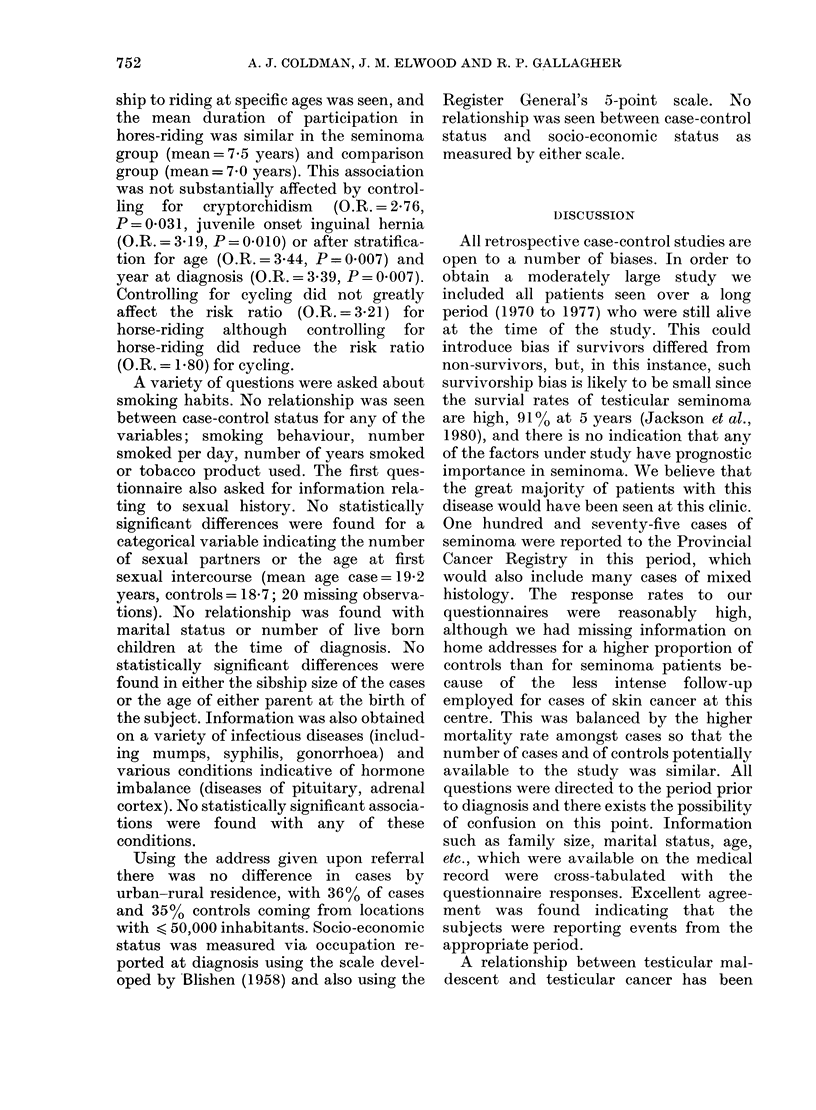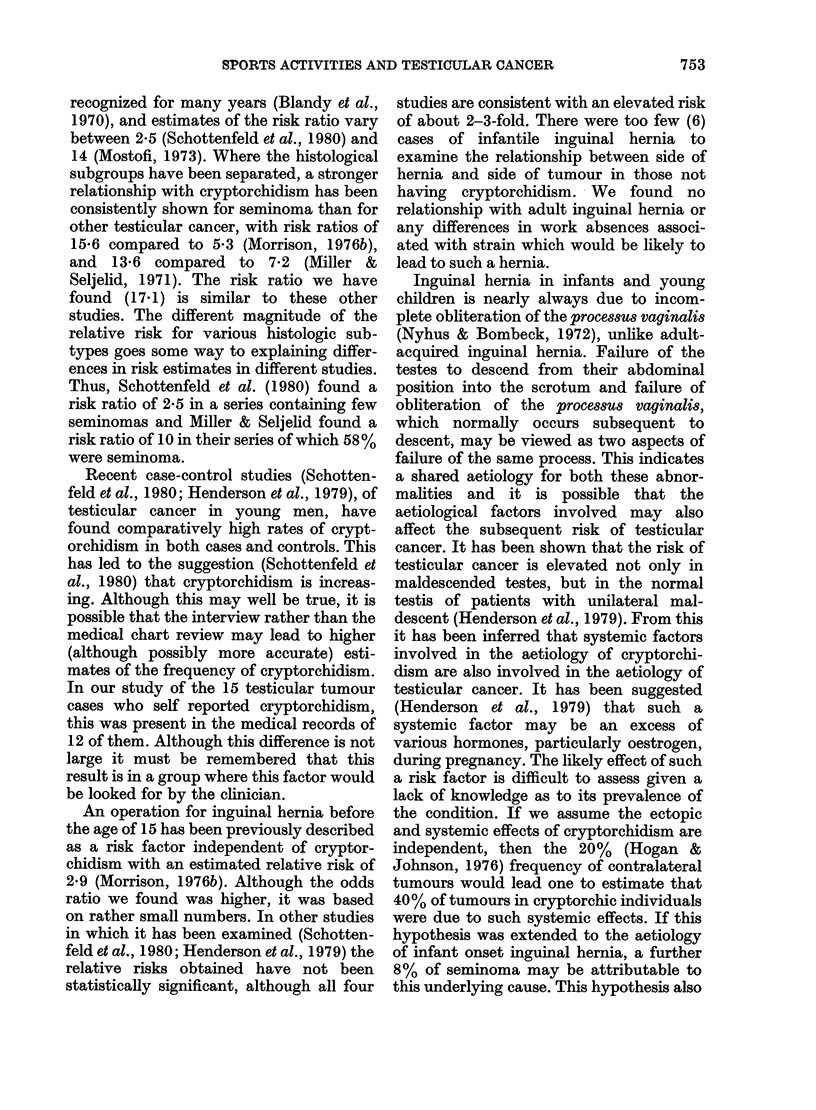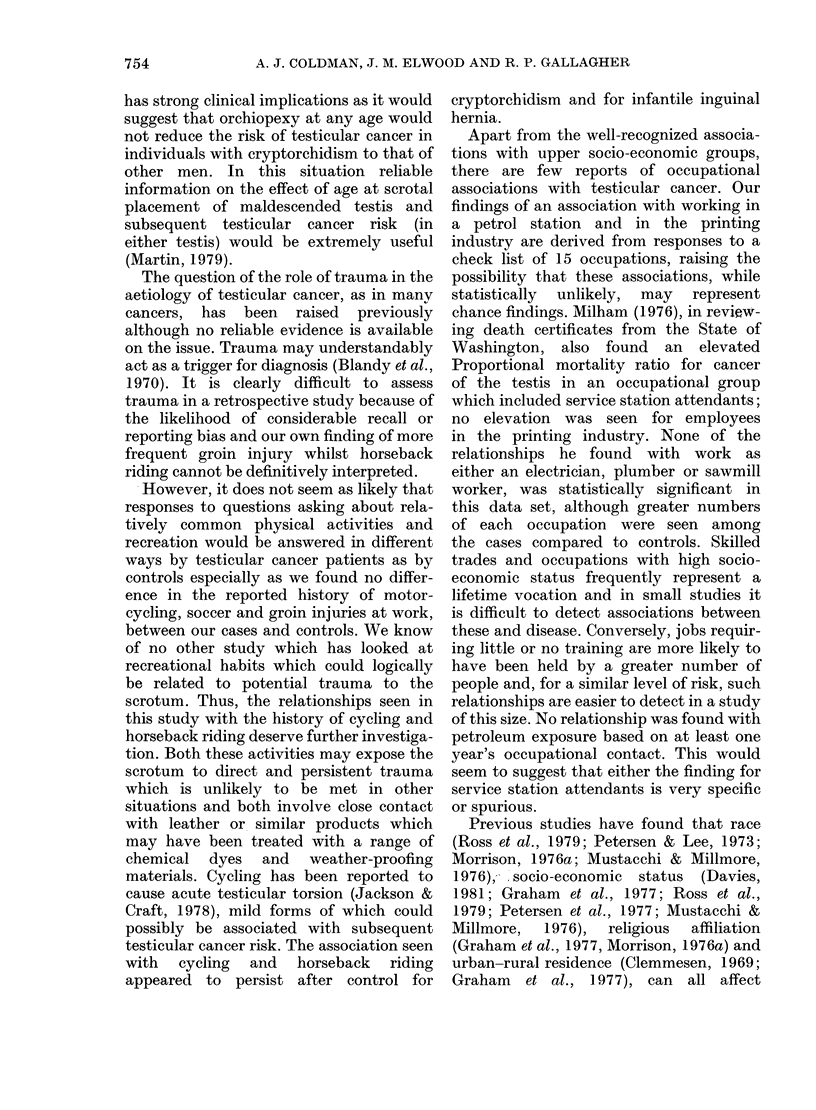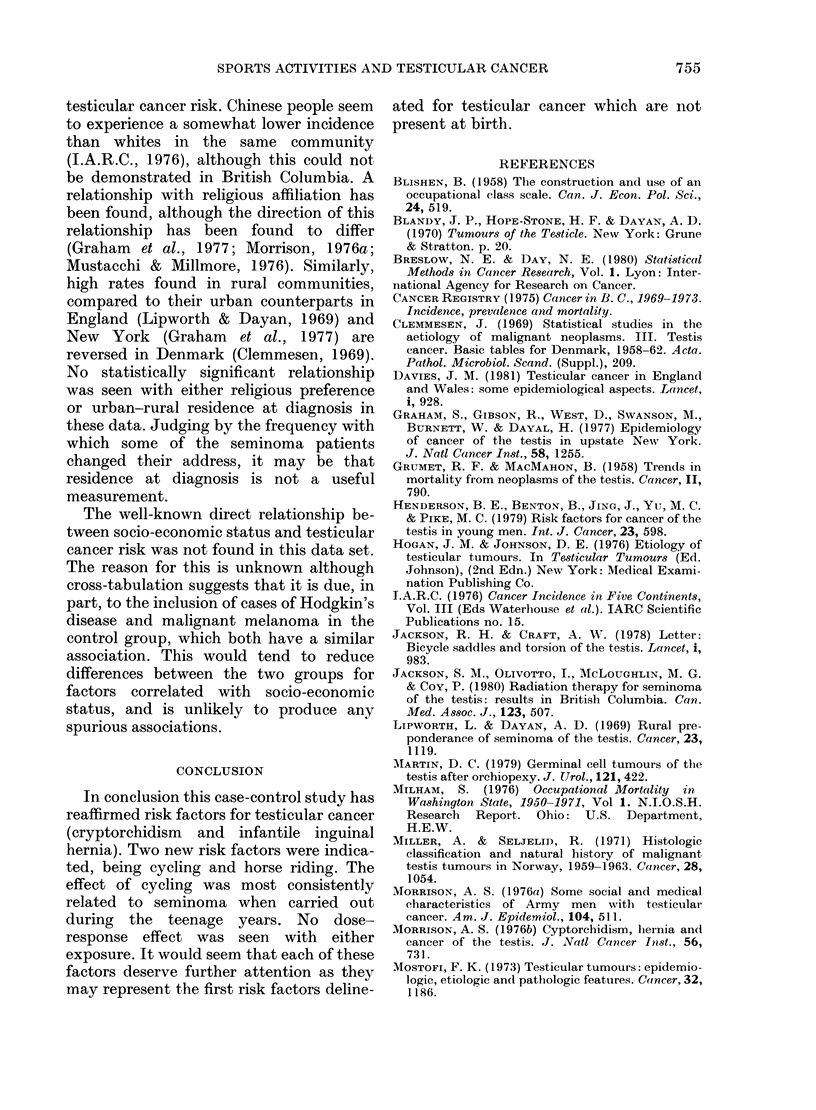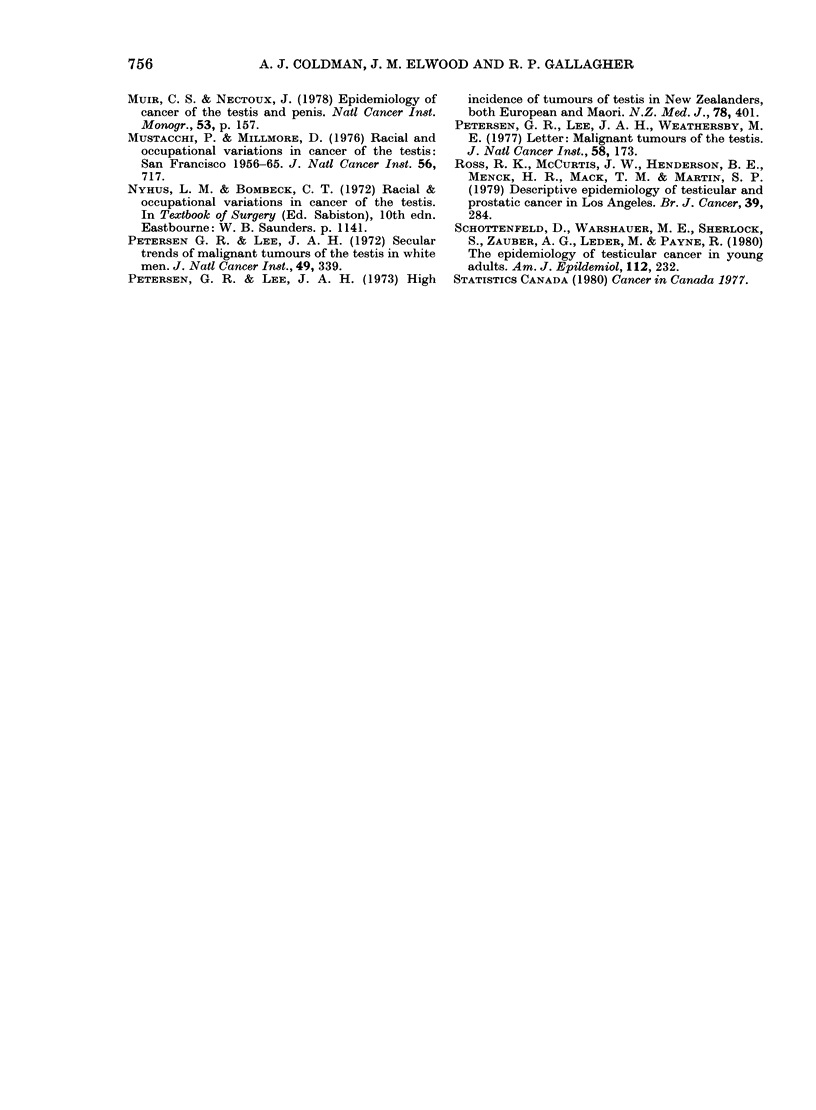# Sports activities and risk of testicular cancer

**DOI:** 10.1038/bjc.1982.267

**Published:** 1982-11

**Authors:** A. J. Coldman, J. M. Elwood, R. P. Gallagher

## Abstract

The relationship of testicular seminoma with several factors was explored using a case-control study. Previously recognized associations with cryptorchidism and infantile inguinal hernia were confirmed and relationships were also found with cycling and horse-riding. These findings represent the first relationships of testicular cancer with well-defined postnatal risk factors.


*Br. J. Cancer* (1982) **46**, 749

### SPORTS ACTIVITIES AND RISK OF TESTICULAR CANCER

A. J. COLDMAN, J. M. ELWOOD[a] AND R. P. GALLAGHER

*From the Department of Epidemiology and Biometry, Cancer Control Agency of B.C., Vancouver, Canada, and [a]the Department of Community Health, Queen's Medical Centre, University of Nottingham, Nottingham*

Received 22 April 1982 Accepted 27 July 1982

**Summary.—The relationship of testicular seminoma with several factors was explored using a case-control study. Previously recognized associations with cryptorchidism and infantile inguinal hernia were confirmed and relationships were also found with cycling and horse-riding. These findings represent the first relationships of testicular cancer with well-defined postnatal risk factors.**

TESTICULAR CANCER is now the most common neoplasm in men aged 25–34 in England and Wales (Davies, 1981) and follows only non-melanoma skin cancer in Canada (Statistics Canada, 1980). Mortality in young men has been rising during this century in a number of countries (Davies, 1981; Grumet & MacMahon, 1958). Similar increases in incidence have been reported by several cancer registries for the age range 15–30 (Muir & Nectoux, 1978; Schottenfeld *et al.*, 1980; Clemmesen, 1969; Petersen & Lee, 1972). Current estimates of incidence in British Columbia indicate that approximately 1 in 400 males will develop testicular cancer (Cancer Registry, 1975).

The majority of studies of the epidemiology of testicular tumours have used routinely collected data to analyse the effects of factors such as social class, marital status, geography, racial group and religious preference. Case-control studies have analysed factors such as cryptorchidism and other prenatal conditions. Where it was examined, these studies have shown that, while differences exist in the age-specific incidence, treatment and prognosis of seminoma compared to other types of testicular cancer, the relationship to known risk factors is similar (Morrison, 1976*b*; Graham *et al.*, 1977). The present report examines a number of characteristics of childhood and adolescence in a case-control study of all patients treated for seminoma of the testis at a regional treatment centre in the period 1970–77.

### METHODS AND MATERIALS

One hundred and twenty-eight of 133 patients with testicular seminoma, seen at the A. Maxwell Evans Clinic in Vancouver, British Columbia, between 1970 and 1977, were included in the study. Treatment of this disease consists of resection followed by radiotherapy, for which this Centre is the sole source on the mainland of British Columbia. All pathology was reviewed routinely at the Centre by a small group of pathologists and only patients with seminoma (with no other histological types noted) were included. For each patient an age ($\pm 2$ years) and year of diagnosis ($\pm 1$ year) matched male control was chosen from other individuals seen at the same institution with a primary diagnosis of skin cancer (excluding those located on the penis or scrotum) or Hodgkin's disease. The control group comprised of 16 cases of malignant melanoma, 86 with other skin cancer and 26 with Hodgkin's disease.

Information was collected in 3 stages. The medical record of each of the 128 cases and controls was reviewed by one clerk who completed an abstract on each. Current addresses were available for 115 (90%) cases and 113 (88%) controls; 11 cases and 4 controls were known to be deceased.

A questionnaire was sent in July 1979 to the 228 men whose address was known and completed responses were received from 93 (81%) cases and 90 (80%) controls. A second and more detailed questionnaire was sent in October 1980 to each of the respondents to the first questionnaire. Complete responses were obtained from 83 (89%) cases and 79 (88%) controls.

Although data were collected on a matched-pair basis, this did not reflect the stratified nature of the sampling plan. The data were analysed in an unmatched fashion in order to avoid loss of information when one member of a pair did not respond. This decreased the likelihood of detecting differences as the resulting risk ratio is then conservatively biased. In cases of special interest or where the initial findings were suggestive, the data were analysed stratifying for other variables. Tests of significance were calculated using $\chi^2$ tests, with continuity correction for the $2 \times 2$ case. In situations where analysis was made whilst controlling for other factors, asymptotic maximum likelihood estimates of the odds ratio were calculated and tests of significance were made with the Mantel–Haenszel statistic (Breslow & Day, 1980).

### RESULTS

Analysis of the responders to the first questionnaire and to both questionnaires showed no differences in the age distribution or year of diagnosis distribution between case and comparison patients.

In response to the questionnaire, 15/93 seminoma patients reported having had an undescended testis, compared to 1/90 controls, yielding an odds ratio of 17·12 ($P < 0·001$) (Table I). The medical records recorded the undescent in 10 of these seminoma patients, but the one instance of maldescent in the comparison subjects was not recorded. Of the 16 seminoma patients recorded on the medical record as having maldescent, 4 were bilateral and of the 12 unilateral cases the tumour was on the corresponding side in 11. Of the 12 unilaterals, surgical correction had been performed in 5 and hormonal therapy resulted in descent in one case. In one bilateral patient surgery was performed only on one side, in one unilateral patient surgery failed, and in another, spontaneous descent occurred at age 30. In the remaining 7 patients no intervention was recorded.

Table I shows that a significantly elevated risk was seen in those who self-reported a history of inguinal hernia. In those without a history of cryptorchidism the relative risk was lower (Table I) but if cryptorchidism was controlled for, the overall risk was still elevated (O.R. = 2·91, $P = 0·058$). The effect of the inguinal hernia was found to vary strongly with age at diagnosis with those diagnosed before age 15 having a much higher risk (O.R. = 7·62, $P = 0·074$) than those diagnosed after age 15 (O.R. = 1·89, $P = 0·438$) after controlling for cryptorchidism. No consistent information was available from the medical record as to the age of diagnosis of inguinal hernia. For 3 cases

TABLE I.—*The numbers, odds ratio, two-sided probability values and 95% confidence levels for various factors in relation to testicular seminoma*

| | Number of cases | Number of controls | Odds ratio | Probability value | 95% confidence interval |
|---|---|---|---|---|---|
| Cryptorchidism (MR) | 16 | 0 | ∞ | 0·0001 | (4·66, ∞) |
| Cryptorchidism (Q1) | 15 | 1 | 17·12 | 0·0009 | (2·28, 365·82) |
| Inguinal hernia (MR) | 25 | 10 | 2·86 | 0·011 | (1·24, 6·74) |
| Inguinal hernia (Q1) | 19 | 6 | 3·60 | 0·013 | (1·27, 10·69) |
| Inguinal hernia with no cryptorchidism (Q1) | 13 | 6 | 2·77 | 0·077 | (0·91, 8·70) |
| Ever worked at a filling station (Q1) | 18 | 6 | 3·36 | 0·021 | (1·18, 10·05) |
| Ever worked in the printing industry (Q1) | 7 | 1 | 7·24 | 0·079 | (0·87, 162·25) |

MR refers to the medical record and Q1 to the first questionnaire as sources. When the estimate of the odds is infinite, one-sided intervals are reported.

TABLE II.—*The numbers, odds ratio, two-sided probability values and 95% confidence levels for various factors in relation to testicular seminoma*

| | Number of cases | Number of controls | Odds ratio | Probability value | 95% confidence interval |
|---|---|---|---|---|---|
| Cycling (Q1) | 44 | 28 | 1·99 | 0·037 | (1·04, 3·81) |
| Horse-riding (Q1) | 25 | 9 | 3·31 | 0·006 | (1·36, 8·25) |
| Motorcycling (Q1) | 16 | 15 | 1·04 | 0·919 | (0·43, 2·41) |
| Soccer (Q1) | 18 | 16 | 1·11 | 0·933 | (0·50, 2·50) |
| Cycling and horse-riding (Q1) | 16 | 5 | 4·56 | 0·008 | (1·41, 15·65) |
| Cycling for sport or recreation as a teenager (Q2) | 62 | 49 | 1·81 | 0·118 | (0·88, 3·75) |
| Cycling to school as a teenager (Q2) | 40 | 27 | 1·79 | 0·090 | (0·91, 3·55) |
| Frequent horse-riding (Q2) | 21 | 11 | 2·09 | 0·106 | (0·88, 5·08) |
| Groin injury whilst horse-riding (Q2) | 5 | 0 | ∞ | 0·079 | (1·116, ∞) |

Q1 refers to the first questionnaire and Q2 to the second questionnaire as the sources of information. When the odds ratio is infinite, one-sided confidence intervals are reported.

and one control the inguinal hernia reported on the questionnaire was not noted on the medical record.

A detailed occupational history was obtained for all jobs held in excess of one year and respondents were also asked to indicate on a check list whether they had ever worked in any of the following industries or occupations: oil drilling or exploration; oil refining; petroleum filling station; rubber or plastic manufacture; highway paving; dyeing or dye manufacture; mining; leather tanning; printing; truck driving; blast furnace; motor mechanic or metal machinist. Elevated risks were associated with work at a filling station and work in the printing industry (Table II). History of work as a plumber or pipefitter, woodworker or sawmill worker, electrician and teacher were also compared by case control status but no statistically significant differences were found.

Using the occupational history information, 4 indices were developed for each of a number of possibly carcinogenic substances or exposures. The first two of these were based on the occupation reported by the subject and the second two on the industry in which he worked. For each pair of indices, one was categorical and indicated ever/never exposure and the other reflected accumulated exposure time. Indices were developed for exposure to radiation, petroleum products, plastic or rubber processing, inks and dyes and pesticides. None of these measures showed significant differences between seminoma patients and controls.

Questions were asked about regular sports and physical recreation, with particular reference to cycling, horseback riding, motorcycling and soccer (Table II). No associations were seen with motorcycling or soccer. The relationships seen with cycling was maintained after controlling for cryptorchidism (O.R. = 1·91, $P = 0{\cdot}062$), juvenile onset inguinal hernia (O.R. = 1·85, $P = 0{\cdot}070$) and after stratification for age (O.R. = 1·98, $P = 0{\cdot}044$) and year at diagnosis (O.R. = 1·96, $P = 0{\cdot}048$). The second questionnaire asked about cycling at different periods of life. No statistically significant associations were found for any aspect of cycling. However, a consistent pattern of elevated risks was seen for various measures related to cycling as a teenager (Table II). Risk ratios for the same variables at other periods of life were all close to unity.

Participation in horse-riding was reported by 25 seminoma patients and 9 comparison subjects, giving an odds ratio of 3·31 ($P = 0{\cdot}006$). A similar question repeated in the second questionnaire defined participation as at least once per month for one year or more, where 21 seminoma patients and 11 comparison patients replied affirmatively. No relationship to riding at specific ages was seen, and the mean duration of participation in hores-riding was similar in the seminoma group (mean = 7·5 years) and comparison group (mean = 7·0 years). This association was not substantially affected by controlling for cryptorchidism (O.R. = 2·76, $P = 0{\cdot}031$, juvenile onset inguinal hernia (O.R. = 3·19, $P = 0{\cdot}010$) or after stratification for age (O.R. = 3·44, $P = 0{\cdot}007$) and year at diagnosis (O.R. = 3·39, $P = 0{\cdot}007$). Controlling for cycling did not greatly affect the risk ratio (O.R. = 3·21) for horse-riding although controlling for horse-riding did reduce the risk ratio (O.R. = 1·80) for cycling.

A variety of questions were asked about smoking habits. No relationship was seen between case-control status for any of the variables; smoking behaviour, number smoked per day, number of years smoked or tobacco product used. The first questionnaire also asked for information relating to sexual history. No statistically significant differences were found for a categorical variable indicating the number of sexual partners or the age at first sexual intercourse (mean age case = 19·2 years, controls = 18·7; 20 missing observations). No relationship was found with marital status or number of live born children at the time of diagnosis. No statistically significant differences were found in either the sibship size of the cases or the age of either parent at the birth of the subject. Information was also obtained on a variety of infectious diseases (including mumps, syphilis, gonorrhoea) and various conditions indicative of hormone imbalance (diseases of pituitary, adrenal cortex). No statistically significant associations were found with any of these conditions.

Using the address given upon referral there was no difference in cases by urban–rural residence, with 36% of cases and 35% controls coming from locations with $\leqslant$ 50,000 inhabitants. Socio-economic status was measured via occupation reported at diagnosis using the scale developed by Blishen (1958) and also using the Register General's 5-point scale. No relationship was seen between case-control status and socio-economic status as measured by either scale.

### DISCUSSION

All retrospective case-control studies are open to a number of biases. In order to obtain a moderately large study we included all patients seen over a long period (1970 to 1977) who were still alive at the time of the study. This could introduce bias if survivors differed from non-survivors, but, in this instance, such survivorship bias is likely to be small since the survial rates of testicular seminoma are high, 91% at 5 years (Jackson *et al.*, 1980), and there is no indication that any of the factors under study have prognostic importance in seminoma. We believe that the great majority of patients with this disease would have been seen at this clinic. One hundred and seventy-five cases of seminoma were reported to the Provincial Cancer Registry in this period, which would also include many cases of mixed histology. The response rates to our questionnaires were reasonably high, although we had missing information on home addresses for a higher proportion of controls than for seminoma patients because of the less intense follow-up employed for cases of skin cancer at this centre. This was balanced by the higher mortality rate amongst cases so that the number of cases and of controls potentially available to the study was similar. All questions were directed to the period prior to diagnosis and there exists the possibility of confusion on this point. Information such as family size, marital status, age, *etc.*, which were available on the medical record were cross-tabulated with the questionnaire responses. Excellent agreement was found indicating that the subjects were reporting events from the appropriate period.

A relationship between testicular maldescent and testicular cancer has been recognized for many years (Blandy *et al.*, 1970), and estimates of the risk ratio vary between 2·5 (Schottenfeld *et al.*, 1980) and 14 (Mostofi, 1973). Where the histological subgroups have been separated, a stronger relationship with cryptorchidism has been consistently shown for seminoma than for other testicular cancer, with risk ratios of 15·6 compared to 5·3 (Morrison, 1976*b*), and 13·6 compared to 7·2 (Miller & Seljelid, 1971). The risk ratio we have found (17·1) is similar to these other studies. The different magnitude of the relative risk for various histologic subtypes goes some way to explaining differences in risk estimates in different studies. Thus, Schottenfeld *et al.* (1980) found a risk ratio of 2·5 in a series containing few seminomas and Miller & Seljelid found a risk ratio of 10 in their series of which 58% were seminoma.

Recent case-control studies (Schottenfeld *et al.*, 1980; Henderson *et al.*, 1979), of testicular cancer in young men, have found comparatively high rates of cryptorchidism in both cases and controls. This has led to the suggestion (Schottenfeld *et al.*, 1980) that cryptorchidism is increasing. Although this may well be true, it is possible that the interview rather than the medical chart review may lead to higher (although possibly more accurate) estimates of the frequency of cryptorchidism. In our study of the 15 testicular tumour cases who self reported cryptorchidism, this was present in the medical records of 12 of them. Although this difference is not large it must be remembered that this result is in a group where this factor would be looked for by the clinician.

An operation for inguinal hernia before the age of 15 has been previously described as a risk factor independent of cryptorchidism with an estimated relative risk of 2·9 (Morrison, 1976*b*). Although the odds ratio we found was higher, it was based on rather small numbers. In other studies in which it has been examined (Schottenfeld *et al.*, 1980; Henderson *et al.*, 1979) the relative risks obtained have not been statistically significant, although all four studies are consistent with an elevated risk of about 2–3-fold. There were too few (6) cases of infantile inguinal hernia to examine the relationship between side of hernia and side of tumour in those not having cryptorchidism. We found no relationship with adult inguinal hernia or any differences in work absences associated with strain which would be likely to lead to such a hernia.

Inguinal hernia in infants and young children is nearly always due to incomplete obliteration of the *processus vaginalis* (Nyhus & Bombeck, 1972), unlike adult-acquired inguinal hernia. Failure of the testes to descend from their abdominal position into the scrotum and failure of obliteration of the *processus vaginalis*, which normally occurs subsequent to descent, may be viewed as two aspects of failure of the same process. This indicates a shared aetiology for both these abnormalities and it is possible that the aetiological factors involved may also affect the subsequent risk of testicular cancer. It has been shown that the risk of testicular cancer is elevated not only in maldescended testes, but in the normal testis of patients with unilateral maldescent (Henderson *et al.*, 1979). From this it has been inferred that systemic factors involved in the aetiology of cryptorchidism are also involved in the aetiology of testicular cancer. It has been suggested (Henderson *et al.*, 1979) that such a systemic factor may be an excess of various hormones, particularly oestrogen, during pregnancy. The likely effect of such a risk factor is difficult to assess given a lack of knowledge as to its prevalence of the condition. If we assume the ectopic and systemic effects of cryptorchidism are independent, then the 20% (Hogan & Johnson, 1976) frequency of contralateral tumours would lead one to estimate that 40% of tumours in cryptorchic individuals were due to such systemic effects. If this hypothesis was extended to the aetiology of infant onset inguinal hernia, a further 8% of seminoma may be attributable to this underlying cause. This hypothesis also has strong clinical implications as it would suggest that orchiopexy at any age would not reduce the risk of testicular cancer in individuals with cryptorchidism to that of other men. In this situation reliable information on the effect of age at scrotal placement of maldescended testis and subsequent testicular cancer risk (in either testis) would be extremely useful (Martin, 1979).

The question of the role of trauma in the aetiology of testicular cancer, as in many cancers, has been raised previously although no reliable evidence is available on the issue. Trauma may understandably act as a trigger for diagnosis (Blandy *et al.*, 1970). It is clearly difficult to assess trauma in a retrospective study because of the likelihood of considerable recall or reporting bias and our own finding of more frequent groin injury whilst horseback riding cannot be definitively interpreted.

However, it does not seem as likely that responses to questions asking about relatively common physical activities and recreation would be answered in different ways by testicular cancer patients as by controls especially as we found no difference in the reported history of motorcycling, soccer and groin injuries at work, between our cases and controls. We know of no other study which has looked at recreational habits which could logically be related to potential trauma to the scrotum. Thus, the relationships seen in this study with the history of cycling and horseback riding deserve further investigation. Both these activities may expose the scrotum to direct and persistent trauma which is unlikely to be met in other situations and both involve close contact with leather or similar products which may have been treated with a range of chemical dyes and weather-proofing materials. Cycling has been reported to cause acute testicular torsion (Jackson & Craft, 1978), mild forms of which could possibly be associated with subsequent testicular cancer risk. The association seen with cycling and horseback riding appeared to persist after control for cryptorchidism and for infantile inguinal hernia.

Apart from the well-recognized associations with upper socio-economic groups, there are few reports of occupational associations with testicular cancer. Our findings of an association with working in a petrol station and in the printing industry are derived from responses to a check list of 15 occupations, raising the possibility that these associations, while statistically unlikely, may represent chance findings. Milham (1976), in reviewing death certificates from the State of Washington, also found an elevated Proportional mortality ratio for cancer of the testis in an occupational group which included service station attendants; no elevation was seen for employees in the printing industry. None of the relationships he found with work as either an electrician, plumber or sawmill worker, was statistically significant in this data set, although greater numbers of each occupation were seen among the cases compared to controls. Skilled trades and occupations with high socio-economic status frequently represent a lifetime vocation and in small studies it is difficult to detect associations between these and disease. Conversely, jobs requiring little or no training are more likely to have been held by a greater number of people and, for a similar level of risk, such relationships are easier to detect in a study of this size. No relationship was found with petroleum exposure based on at least one year's occupational contact. This would seem to suggest that either the finding for service station attendants is very specific or spurious.

Previous studies have found that race (Ross *et al.*, 1979; Petersen & Lee, 1973; Morrison, 1976*a*; Mustacchi & Millmore, 1976), socio-economic status (Davies, 1981; Graham *et al.*, 1977; Ross *et al.*, 1979; Petersen *et al.*, 1977; Mustacchi & Millmore, 1976), religious affiliation (Graham *et al.*, 1977, Morrison, 1976*a*) and urban–rural residence (Clemmesen, 1969; Graham *et al.*, 1977), can all affect testicular cancer risk. Chinese people seem to experience a somewhat lower incidence than whites in the same community (I.A.R.C., 1976), although this could not be demonstrated in British Columbia. A relationship with religious affiliation has been found, although the direction of this relationship has been found to differ (Graham *et al.*, 1977; Morrison, 1976*a*; Mustacchi & Millmore, 1976). Similarly, high rates found in rural communities, compared to their urban counterparts in England (Lipworth & Dayan, 1969) and New York (Graham *et al.*, 1977) are reversed in Denmark (Clemmesen, 1969). No statistically significant relationship was seen with either religious preference or urban–rural residence at diagnosis in these data. Judging by the frequency with which some of the seminoma patients changed their address, it may be that residence at diagnosis is not a useful measurement.

The well-known direct relationship between socio-economic status and testicular cancer risk was not found in this data set. The reason for this is unknown although cross-tabulation suggests that it is due, in part, to the inclusion of cases of Hodgkin's disease and malignant melanoma in the control group, which both have a similar association. This would tend to reduce differences between the two groups for factors correlated with socio-economic status, and is unlikely to produce any spurious associations.

### CONCLUSION

In conclusion this case-control study has reaffirmed risk factors for testicular cancer (cryptorchidism and infantile inguinal hernia). Two new risk factors were indicated, being cycling and horse riding. The effect of cycling was most consistently related to seminoma when carried out during the teenage years. No dose–response effect was seen with either exposure. It would seem that each of these factors deserve further attention as they may represent the first risk factors delineated for testicular cancer which are not present at birth.

### REFERENCES

BLISHEN, B. (1958) The construction and use of an occupational class scale. *Can. J. Econ. Pol. Sci.*, **24**, 519.

BLANDY, J. P., HOPE-STONE, H. F. & DAYAN, A. D. (1970) *Tumours of the Testicle.* New York: Grune & Stratton. p. 20.

BRESLOW, N. E. & DAY, N. E. (1980) *Statistical Methods in Cancer Research*, Vol. **1**. Lyon: International Agency for Research on Cancer.

CANCER REGISTRY (1975) *Cancer in B. C., 1969–1973. Incidence, prevalence and mortality.*

CLEMMESEN, J. (1969) Statistical studies in the aetiology of malignant neoplasms. III. Testis cancer. Basic tables for Denmark, 1958–62. *Acta. Pathol. Microbiol. Scand.* (Suppl.), 209.

DAVIES, J. M. (1981) Testicular cancer in England and Wales: some epidemiological aspects. *Lancet*, **i**, 928.

GRAHAM, S., GIBSON, R., WEST, D., SWANSON, M., BURNETT, W. & DAYAL, H. (1977) Epidemiology of cancer of the testis in upstate New York. *J. Natl Cancer Inst.*, **58**, 1255.

GRUMET, R. F. & MACMAHON, B. (1958) Trends in mortality from neoplasms of the testis. *Cancer*, **II**, 790.

HENDERSON, B. E., BENTON, B., JING, J., YU, M. C. & PIKE, M. C. (1979) Risk factors for cancer of the testis in young men. *Int. J. Cancer*, **23**, 598.

HOGAN, J. M. & JOHNSON, D. E. (1976) Etiology of testicular tumours. In *Testicular Tumours* (Ed. Johnson), (2nd Edn.) New York: Medical Examination Publishing Co.

I.A.R.C. (1976) *Cancer Incidence in Five Continents*, Vol. III (Eds Waterhouse *et al.*). IARC Scientific Publications no. 15.

JACKSON, R. H. & CRAFT, A. W. (1978) Letter: Bicycle saddles and torsion of the testis. *Lancet*, **i**, 983.

JACKSON, S. M., OLIVOTTO, I., MCLOUGHLIN, M. G. & COY, P. (1980) Radiation therapy for seminoma of the testis: results in British Columbia. *Can. Med. Assoc. J.*, **123**, 507.

LIPWORTH, L. & DAYAN, A. D. (1969) Rural preponderance of seminoma of the testis. *Cancer*, **23**, 1119.

MARTIN, D. C. (1979) Germinal cell tumours of the testis after orchiopexy. *J. Urol.*, **121**, 422.

MILHAM, S. (1976) *Occupational Mortality in Washington State, 1950–1971*, Vol **1**. N.I.O.S.H. Research Report. Ohio: U.S. Department, H.E.W.

MILLER, A. & SELJELID, R. (1971) Histologic classification and natural history of malignant testis tumours in Norway, 1959–1963. *Cancer*, **28**, 1054.

MORRISON, A. S. (1976*a*) Some social and medical characteristics of Army men with testicular cancer. *Am. J. Epidemiol.*, **104**, 511.

MORRISON, A. S. (1976*b*) Cyptorchidism, hernia and cancer of the testis. *J. Natl Cancer Inst.*, **56**, 731.

MOSTOFI, F. K. (1973) Testicular tumours: epidemiologic, etiologic and pathologic features. *Cancer*, **32**, 1186.

MUIR, C. S. & NECTOUX, J. (1978) Epidemiology of cancer of the testis and penis. *Natl Cancer Inst. Monogr.*, **53**, p. 157.

MUSTACCHI, P. & MILLMORE, D. (1976) Racial and occupational variations in cancer of the testis: San Francisco 1956–65. *J. Natl Cancer Inst.* **56**, 717.

NYHUS, L. M. & BOMBECK, C. T. (1972) Racial & occupational variations in cancer of the testis. In *Textbook of Surgery* (Ed. Sabiston), 10th edn. Eastbourne: W. B. Saunders. p. 1141.

PETERSEN G. R. & LEE, J. A. H. (1972) Secular trends of malignant tumours of the testis in white men. *J. Natl Cancer Inst.*, **49**, 339.

PETERSEN, G. R. & LEE, J. A. H. (1973) High incidence of tumours of testis in New Zealanders, both European and Maori. *N.Z. Med. J.*, **78**, 401.

PETERSEN, G. R., LEE, J. A. H., WEATHERSBY, M. E. (1977) Letter: Malignant tumours of the testis. *J. Natl Cancer Inst.*, **58**, 173.

ROSS, R. K., MCCURTIS, J. W., HENDERSON, B. E., MENCK, H. R., MACK, T. M. & MARTIN, S. P. (1979) Descriptive epidemiology of testicular and prostatic cancer in Los Angeles. *Br. J. Cancer*, **39**, 284.

SCHOTTENFELD, D., WARSHAUER, M. E., SHERLOCK, S., ZAUBER, A. G., LEDER, M. & PAYNE, R. (1980) The epidemiology of testicular cancer in young adults. *Am. J. Epildemiol*, **112**, 232.

STATISTICS CANADA (1980) *Cancer in Canada 1977.*